# Multilevel Impacts of Habitat Fragmentation on Plant Diversity and Structure in Urban Remnant Forests

**DOI:** 10.1002/ece3.74246

**Published:** 2026-08-28

**Authors:** Linfeng Zhou, Bingxia Zhang, Mengli He, Minglu Guo, Jingyi Yang

**Affiliations:** ^1^ College of Forestry, Institute for Forest Resources and Environment of Guizhou, Guizhou Key Laboratory of Forest Cultivation in Plateau Mountain Guizhou University Guiyang China; ^2^ Guizhou Guiyang Urban Ecosystem Observation and Research Station National Forestry and Grassland Administration Guiyang China

**Keywords:** forest structure, fragmentation, Guiyang, mediation effect, plant diversity, urban remnant forests

## Abstract

Against the backdrop of ongoing global urbanization, understanding the impact of urban remnant forest fragmentation on plant diversity and community structure is crucial for developing effective biodiversity conservation and urban management strategies. This study investigated 30 remnant forest patches in the Guiyang metropolitan area of China, quantifying fragmentation indices at both patch and landscape scales to analyze how plant diversity and forest structure vary along a fragmentation gradient in interior and edge habitats. The results showed that edge habitats exhibited higher forest structural metrics (total basal area, dominant tree height, and total biomass), but their plant diversity was negatively affected by the clumpiness of surrounding impervious surfaces. In interior habitats, diversity was negatively influenced by edge contrast and clumpiness of impervious surface. In addition, forest structure was also negatively affected by the clumpiness of surrounding impervious surfaces and positively affected by patch area. Furthermore, significant positive relationships were detected between tree density and plant diversity, and the composition of understory vegetation (including saplings/seedlings, shrubs, and herbs) was significantly influenced by forest structural attributes. Based on these results, we recommend maintaining patch integrity, establishing buffer zones to reduce edge contrast, and controlling the contiguity of impervious surfaces around patches to sustain both plant diversity and forest structural complexity in urban remnant forests.

## Graphical abstract

This study of 30 urban remnant forest patches in Guiyang, China, found that edge habitats displayed greater forest structural metrics but reduced plant diversity linked to the clumpiness of surrounding impervious surfaces, whereas interior diversity suffered from high edge contrast and impervious clumpiness. Forest structure was also negatively associated with impervious surface contiguity, and understory composition was shaped by structural attributes, leading to recommendations that prioritize patch integrity, buffer zones to soften edge contrast, and control of contiguous impervious surfaces to sustain both diversity and structural complexity.

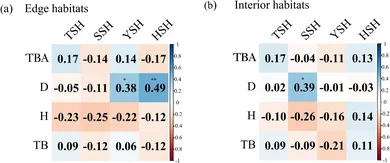

## 1 Introduction

Urbanization exerts substantial pressure on global ecosystems and represents a major driver of biodiversity loss (Silva‐Junior et al. 2018). Meanwhile, China, which is currently undergoing rapid urbanization, has experienced extensive fragmentation of its formerly continuous forest landscapes due to the expansion of infrastructure such as roads and buildings. This has resulted in the formation of isolated forest patches. These remnant urban forests serve as critical and scarce habitat units that play a vital role in maintaining regional biodiversity. Furthermore, in addition to their irreplaceable functions in conserving biodiversity, mitigating the urban heat island effect, and improving air quality (Chen et al. 2021), remnant forests provide essential ecosystem services that enhance the living environment for urban residents (Foo 2016).

However, ongoing urban expansion poses severe threats to these ecosystems. Urban sprawl leads to the loss of remnant forest habitat and alters the structure and function of the remaining forest patches (Yang et al. 2024; Zheng and Yang 2023). Although some fragmented habitats may serve as temporary refuges for certain species or even exhibit high species richness at local scales, their negative impacts remain dominant from landscape ecological and population genetic perspectives (Holderegger and Di Giulio 2010; Rasmussen et al. 2020). Habitat fragmentation directly reduces effective habitat area for species, intensifies edge effects, and diminishes core habitat areas (Pfeifer et al. 2017). This fragmentation typically results in reduced population sizes, increased local extinction rates, and ultimately leads to decreased regional species richness (Estavillo et al. 2013; Zhang et al. 2022). Furthermore, habitat fragmentation reshapes interspecific relationships and alters crucial ecological processes such as competition, predation, pollination, and seed dispersal, thereby increasing extinction risks for specialized species with limited adaptive capacity (Haddad et al. 2015; Tee et al. 2018; Vranckx et al. 2012).

Forest fragmentation influences forest structure through multiple mechanisms. At the landscape level, continuous forest habitats are divided into isolated patches (Haddad et al. 2015), leading to an increased edge‐to‐interior ratio, reduced core habitat area (Fischer et al. 2021), and altered microclimatic conditions such as temperature, humidity, and light availability (Yuan et al. 2024). These changes trigger structural adjustments in vegetation, including greater canopy openness, reduced tree layer coverage, and often more developed understory vegetation. Consequently, species composition is reassembled, typically characterized by the decline of interior species and the expansion of generalist or edge‐adapted species, hence diminishing both structural complexity and ecological function of the community (Laurance et al. 2018).

The relationship between species diversity and forest structure has been well investigated in natural forest ecosystems. However, in the context of urban remnant forests, prior studies have predominantly focused on the isolated effects of urbanization on either species diversity or forest structure, while largely overlooking the interrelationships between these two dimensions. In early successional environments shaped by urbanization, such as secondary forests or forest gaps, open habitats and enhanced resource availability amplify niche complementarity effects. Under these conditions, higher species diversity can enhance community productivity through spatiotemporal resource partitioning and complementarity, which in turn accelerates canopy development and increasing vertical structural complexity, key processes that advance forest maturation (Williams et al. 2017). Furthermore, species richness promotes canopy structural complexity via functional diversity, manifested through variations in traits such as tree height, shade tolerance, and wood density, which is reflected in more pronounced vertical stratification, crown morphological differentiation, and spatial complementarity (Xu et al. 2022; Zhang et al. 2022). Given that urban remnant forests are typically small, isolated, and heavily influenced by edge effects and anthropogenic pressures, the diversity–structure relationships established in natural forests may not directly apply, necessitating dedicated investigation in fragmented urban settings.

This study investigates 30 remnant forest patches with varying fragmentation levels in Guiyang, China, to assess the effects of fragmentation on species diversity and forest structure. The specific objectives are: (1) to analyze the impact of remnant forest fragmentation on plant diversity; (2) to identify the effects of fragmentation on forest structure; and (3) to examine the relationship between plant diversity and forest structure in fragmented remnants.

## 2 Methods

### 2.1 Study Area

This study focuses on the metropolitan area of Guiyang as its research region. Guiyang is situated in the central part of Guizhou Province, China, on the eastern slope of the Yunnan–Guizhou Plateau, within the watershed area separating the Wu River of the Yangtze River system and the Hongshui River of the Pearl River system. The region is characterized by typical karst mountains and hills, with over 88% of its land covered by mountainous and hilly terrain. The topography slopes from higher elevations in the southwest to lower areas in the northeast, exhibiting significant altitude variations. This distinctive geographical setting has shaped Guiyang's unique urban landscape, where the city is integrated with mountains, creating a “city within mountains and mountains within the city.” The zonal vegetation consists of evergreen and deciduous broad‐leaved mixed forests, and the dominant soil types are yellow earth and limestone soil. As a typical karst mountain region, Guiyang has preserved considerable areas of urban remnant forests due to the presence of numerous natural hills unsuitable for development, which are embedded within the urban fabric amid rapid urbanization‐driven expansion.

### 2.2 Sample Selection and Field Survey

In the study area, 30 discrete broad‐leaved forest patches ranging in size from nearly 5 to 30 ha and spaced at least 1 km apart were selected as samples. Within each patch, four 20 × 20 m subplots were established along the four cardinal directions: one set located within 25 m of the boundary (edge habitat) and another set at least 100 m from the boundary (interior habitat), resulting in a total of 240 surveyed subplots. This 25 m edge criterion was adopted from previous work in karst landscapes (Wang and Yang 2022) and is supported by studies showing that woodland species composition often changes within 25 m of the boundary (Vallet et al. 2010; Honnay et al. 2002). Although karst topography may alter the penetration of edge effects relative to flat areas, the consistent use of this threshold ensures comparability, while the interior subplots (≥ 100 m from the boundary) provide a robust contrast with minimal edge influence. All distances were measured along the ground surface. Owing to the steep karst terrain, the surface distance is greater than the horizontal distance. In each subplot, all vascular plant species were recorded, including trees, shrubs, and herbs, along with their abundance. Species were classified into growth forms (tree, shrub, or herb) following the Flora of China. For woody species, all individuals of a species defined as a tree were recorded as trees regardless of their size, and then further categorized into adults (diameter at breast height, DBH > 3 cm) and saplings/seedlings (DBH ≤ 3 cm). All individuals of shrub species were recorded as shrubs. This species‐based classification avoids any ambiguity between small tree individuals and shrubs. Additionally, the DBH and tree height of all trees with a DBH ≥ 5 cm were measured. Soil samples were collected from the top layer (0–5 cm depth) after removing surface litter using a five‐point sampling method within each subplot. A uniform 0–5 cm sampling depth was adopted because bedrock frequently restricted extractable soil to this depth in nearly one‐quarter of the patches, and consistent sampling was necessary to ensure comparability of soil properties across all patches. The 0–5 cm layer is the primary zone of nutrient cycling and organic matter accumulation in these karst soils, and it is highly responsive to litter input and microenvironmental changes driven by fragmentation. The soil samples from the four edge subplots and the four interior subplots within the same patch were separately combined, yielding one composite edge sample and one composite interior sample per patch, for a total of 60 soil samples.

### 2.3 Measurement of Soil Property

Following collection, soil samples were air‐dried, and any stones or plant residues were removed. The soil was then passed through a 2 mm sieve to obtain homogeneous analytical samples for determining basic physicochemical properties, including soil organic carbon (SOC), total nitrogen (TN), total phosphorus (TP), and pH. Specifically, SOC was measured using the potassium dichromate volumetric method–dilution heat method, which involves oxidizing organic matter and titrating the remaining oxidant to calculate organic carbon content. TN was determined by the Kjeldahl nitrogen method, in which soil samples are digested and distilled, and nitrogen content is then measured to reflect TN levels (Paltineanu et al. 2024). TP was analyzed by combined digestion with perchloric–sulfuric acid (HClO_4_–H_2_SO_4_), followed by quantitative colorimetric analysis of phosphorus using the molybdenum–antimony anti‐spectrophotometric method (Yu et al. 2024). For pH measurement, soil–water suspensions were prepared at a 1:2.5 ratio and analyzed using the electrode method with a precision pH meter (Fan et al. 2023).

### 2.4 Quantification of Forest Structure

This study employed four key indicators to quantify forest structure: total basal area (TBA), dominant height (*H*), tree density (*D*), and total biomass (TB) (Dupont‐Leduc et al. 2024; Nizami et al. 2017). For each forest patch, data from the four interior or edge subplots were pooled to form a single sample. Accordingly, TBA was calculated as the sum of the basal areas of all trees with a DBH > 5 cm across the four subplots, expressed in m^2^/1600 m^2^. Dominant height (*H*) was determined as the average height of the four tallest trees recorded across the four subplots, reported in meters. Tree density (*D*) represented the total number of trees with DBH > 5 cm in the four subplots, given as individuals/m^2^. TB expressed in t/1600 m^2^, was estimated as the sum of aboveground and belowground biomass, using the following Equations ((1), (2), (3), (4)).

(1) $$M_{A}=a_{0}D^{a_{1}}H^{a_{2}}$$

(2) $$M_{B}={\mathrm{ab}}_{0}D^{b_{1}}H^{b_{2}}$$

(3) $$M_{A}=a_{0}D^{a_{1}}$$

(4) $$M_{B}={\mathrm{ab}}_{0}D^{b_{1}}$$

Equations (1) and (2) represent a two‐variable biomass model for estimating above‐ and belowground biomass, respectively, while Equations (3) and (4) represent a single‐variable biomass model. In these equations, *M*_*A*_ denotes the estimated aboveground biomass in kilograms (kg); *M*_*B*_ denotes the estimated belowground biomass in kilograms (kg); *D* represents the tree diameter at breast height (DBH) in centimeters (cm); and *H* represents the tree height in meters (m). The symbols *a*_0_, *a*_1_, *a*_2_, *b*_0_, *b*_1_, *b*_2_ refer to model parameters, whose specific values for different tree species are provided in Table S1.

The model parameters were sourced from the national standard “Tree Biomass Models and Carbon Accounting Parameters for Major Tree Species” (GB/T 43648–2024), which provides parameters for two‐variable biomass equations of 21 major tree species in China. For tree species that either exactly matched one of the 21 major species or were taxonomically congruent at the genus level, the corresponding two‐variable biomass model from the standard was directly applied. For tree species not included in this list, the general national single‐variable biomass model provided in the standard was used. In cases where a species was neither a major species nor covered by a specific single‐variable model, parameters for general hardwood or softwood models were applied according to the wood properties of the species, ensuring that biomass calculations could be performed for all recorded trees. In our dataset, approximately 65% of individual trees were estimated using the general hardwood or softwood models. This proportion reflects the high tree species diversity encountered in our field survey and the inherently limited number of species‐specific equations available in the national standard. To minimize potential estimation bias, we applied all models exclusively from GB/T 43648–2024, thereby maintaining parameter consistency and avoiding the additional uncertainty that would arise from arbitrarily combining equations from disparate sources.

To quantify the potential bias introduced by applying general hardwood/softwood models to trees lacking species‐specific equations, we performed an internal sensitivity analysis. All trees with species‐ or genus‐level equations (approximately 35% of individuals) were used as validation samples. For each of these trees, biomass was recalculated using the appropriate general hardwood or softwood model. The relative deviation for each individual was obtained by taking the difference between the generic‐model estimate and the species‐specific estimate and dividing it by the species‐specific estimate. The internal validation using trees with species‐specific equations showed that the generic hardwood/softwood models produced a mean relative deviation in individual tree biomass of 32.5%. Thus, the application of generic models did not introduce meaningful systematic bias into our structural metrics.

### 2.5 Selection of Fragmentation Indices

To comprehensively quantify the fragmentation degree of remnant forest patches at both the patch and landscape scales, this study selected eight metrics: Area, Fractal Dimension Index (FRAC), Interior‐to‐Edge Ratio (IER), Edge Contrast Index (ECON), Percentage of Impervious Surface in the surrounding landscape (PIS), Clumpiness Index of Impervious Surface (CLUMPY‐IS), Percentage of Forest Cover in the surrounding landscape (PFC), and Clumpiness Index of Forest Cover (CLUMPY‐FC). These metrics were calculated using Fragstats 4.3 and ArcGIS 10.8. Area directly reflects the degree of habitat loss, with smaller patches generally indicating higher extinction risks. The FRAC, which ranges between 1 and 2, describes shape complexity—higher values correspond to more geometrically complex patch shapes. The IER is defined as the area ratio between the patch interior and its edge zone (within 25 m of the patch boundary). The ECON further quantifies the contrast between the patch edge and adjacent land use types, ranging from 1 to 100; higher ECON values indicate stronger edge effects and greater contrast. The landscape scale was defined as the area within a 1 km radius from the geometric center of each patch. PIS and PFC represent the proportion of impervious surface and forest cover within this zone, respectively, while CLUMPY‐IS and CLUMPY‐FC describe the spatial aggregation of impervious surfaces and forest areas in the same context (Riitters et al. 1996). A 1 km radius buffer scale was selected based on a previous empirical study in Guiyang, which found that landscape indices measured at 1 km consistently captured significant relationships with plant, soil microbe, and multiple ecosystem functions (He et al. 2026). The 1 km radius thus represents an empirically validated, ecologically meaningful spatial extent for this study system.

### 2.6 Statistical Analysis

Statistical analyses were performed using R (version 4.4.3). The nonmetric multidimensional scaling (NMDS) combined with permutational multivariate analysis of variance was used to compare species composition of different plant types between interior and edge habitats. General regression analysis was applied to examine the relationships of plant diversity and four forest structural indicators in remnant forest patches with fragmentation indices and soil properties. The Shannon‐Wiener diversity index was calculated for adult trees (TSH), shrubs (SSH), saplings/seedlings (YSH), and herbs (HSH), respectively, as quantitative measures of plant diversity for each vegetation type. We assessed spatial autocorrelation in the regression response variables (Shannon diversity indices, TBA, biomass, tree density, and height) using Global Moran's *I*. For both edge and interior habitats, no significant spatial autocorrelation was detected for the majority of variables (all *p* > 0.05), except for tree density in the edge habitat, which showed a weak positive spatial structure (Moran's *I* = 0.171, *p* = 0.026; Table S2). Given this overall lack of strong spatial dependence, we used general linear regression models without spatial correction. Paired *t*‐tests were conducted to compare forest structural metrics between interior and edge habitats. Spearman correlation analysis was employed to assess associations between forest structure and plant diversity. Redundancy analysis (RDA) and variance partitioning were used to quantify the relative influences of fragmentation indices, forest structure, and soil properties on the composition of different plant types.

## 3 Results

### 3.1 Effects of Fragmentation and Soil Properties on Plant Diversity and Composition

The NMDS and permutational multivariate analysis revealed no clear separation in species composition of different plant communities between interior and edge habitats (Figure S1). Stress values ranged from 0.208 to 0.275, indicating a moderate goodness‐of‐fit; while not excellent, such values are not uncommon for heterogeneous ecological datasets and are considered tolerable for graphical exploration of broad patterns. Furthermore, the ANOSIM confirmed the absence of significant compositional differences between habitat types (*R*‐values close to 0, all *p* > 0.05).

**FIGURE 1:**
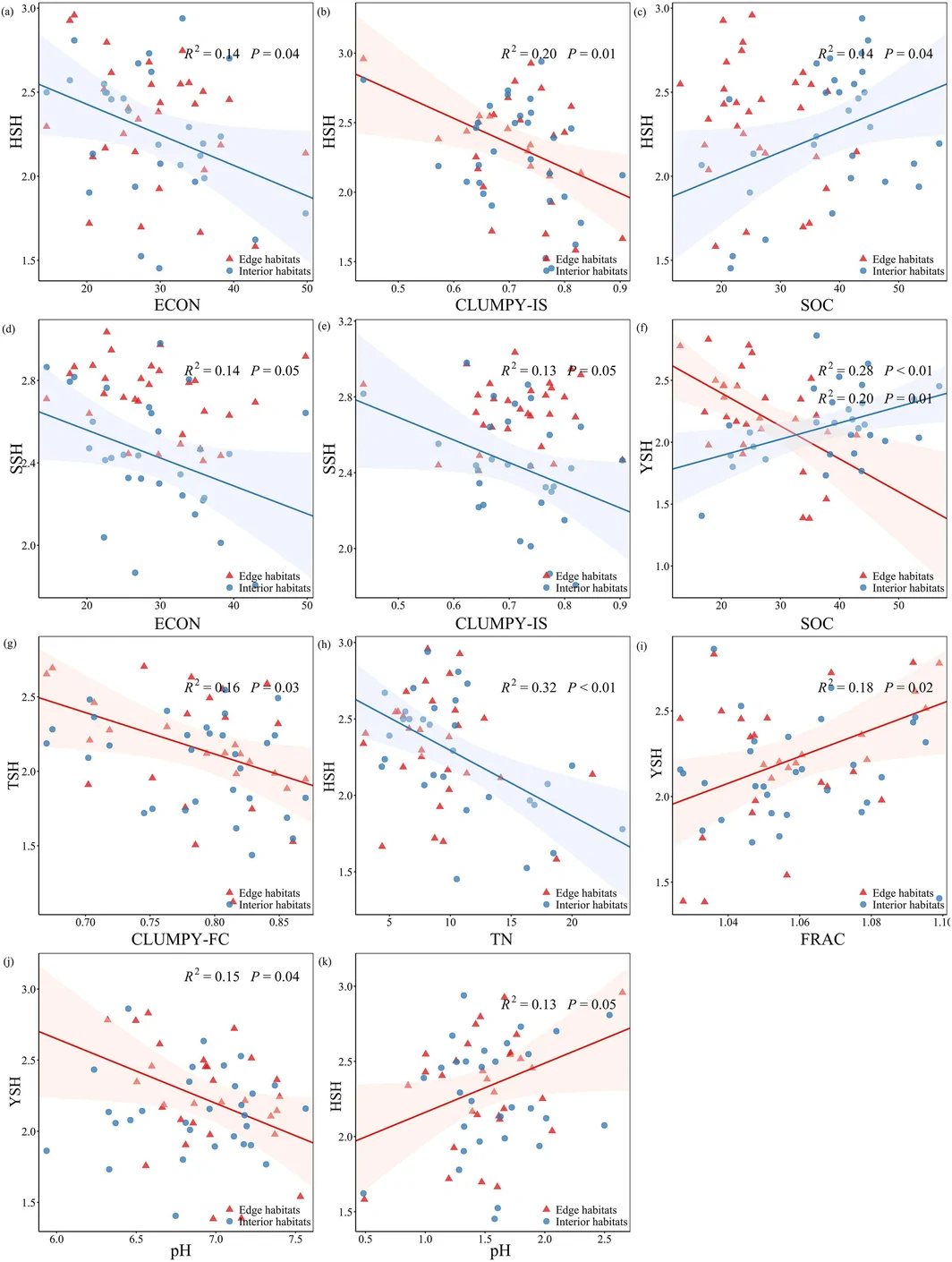
Significant regression relationships between plant diversity and fragmentation indices or soil properties (*p* < 0.05).

Analysis of the factors influencing plant diversity revealed that fragmentation indices and soil properties exerted distinct effects across habitats and plant types. Regarding fragmentation metrics, in the interior habitat, the Shannon diversity of herbs and shrubs showed significant negative correlations with the ECON (Figure 1a,d). The shrub Shannon diversity in the interior was negatively correlated with the CLUMPY‐IS (Figure 1e). In the edge habitat, the Shannon diversity of herbs was negatively correlated with the CLUMPY‐IS (Figure 1b), while the adult tree diversity was negatively associated with the Clumpiness Index of forest cover (Figure 1g). Saplings/seedling diversity at the edge was positively correlated with the FRAC (Figure 1i).

In terms of soil properties, in the interior habitat, the Shannon diversity of herbs was positively correlated with SOC (Figure 1c) but negatively correlated with TN (Figure 1h). Saplings/seedling diversity in the interior was also positively correlated with SOC (Figure 1f). In the edge habitat, saplings/seedling diversity was negatively correlated with both SOC (Figure 1f) and soil pH (Figure 1j), whereas herb diversity was positively correlated with TP (Figure 1k).

### 3.2 Impacts on Remnant Forest Structure

**FIGURE 2:**
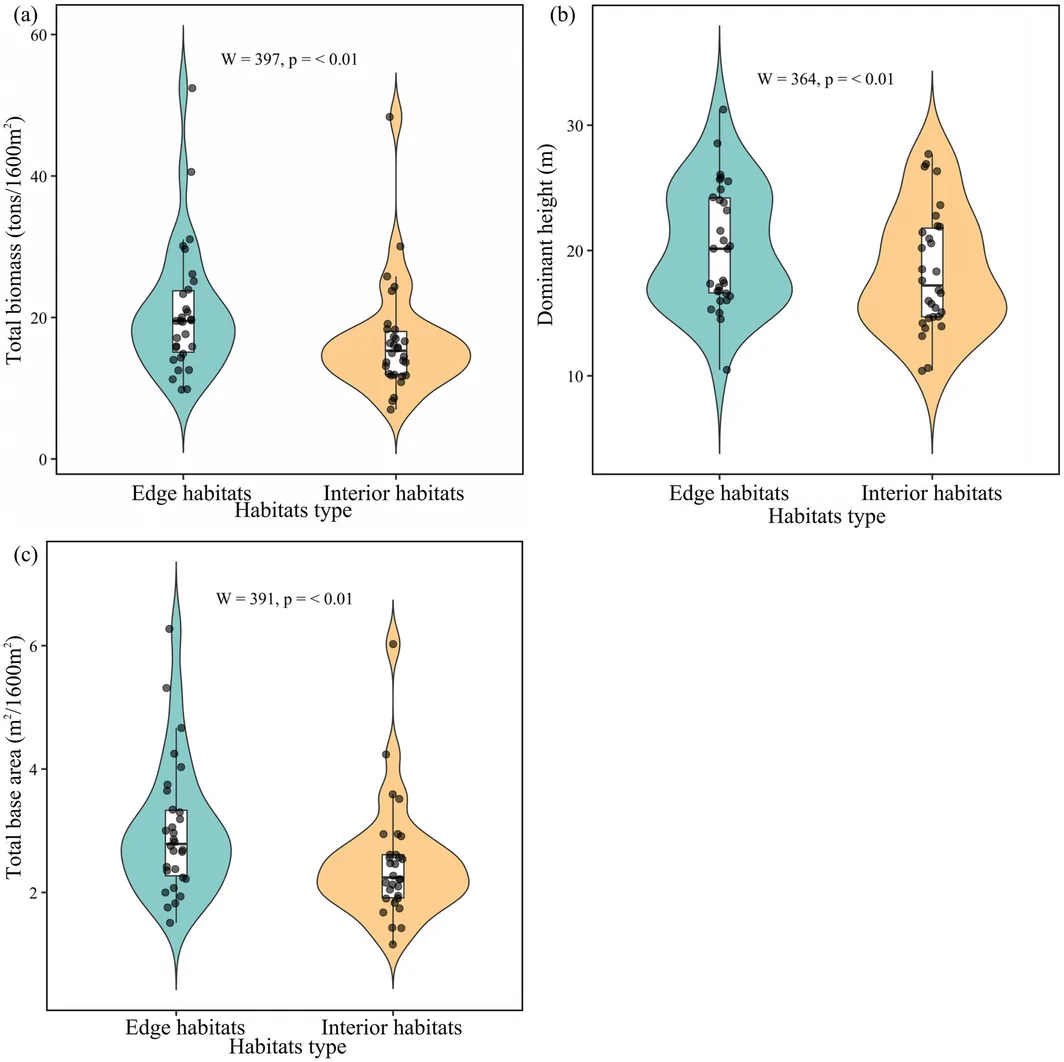
Significant differences in forest structural indices based on *t*‐test comparisons between habitats (*p* < 0.01).

The *t*‐test results comparing forest structural metrics between interior and edge habitats revealed that the TBA, dominant height, and TB were all significantly greater in the edge habitat than in the interior (Figure 2, *p* < 0.01).

**FIGURE 3:**
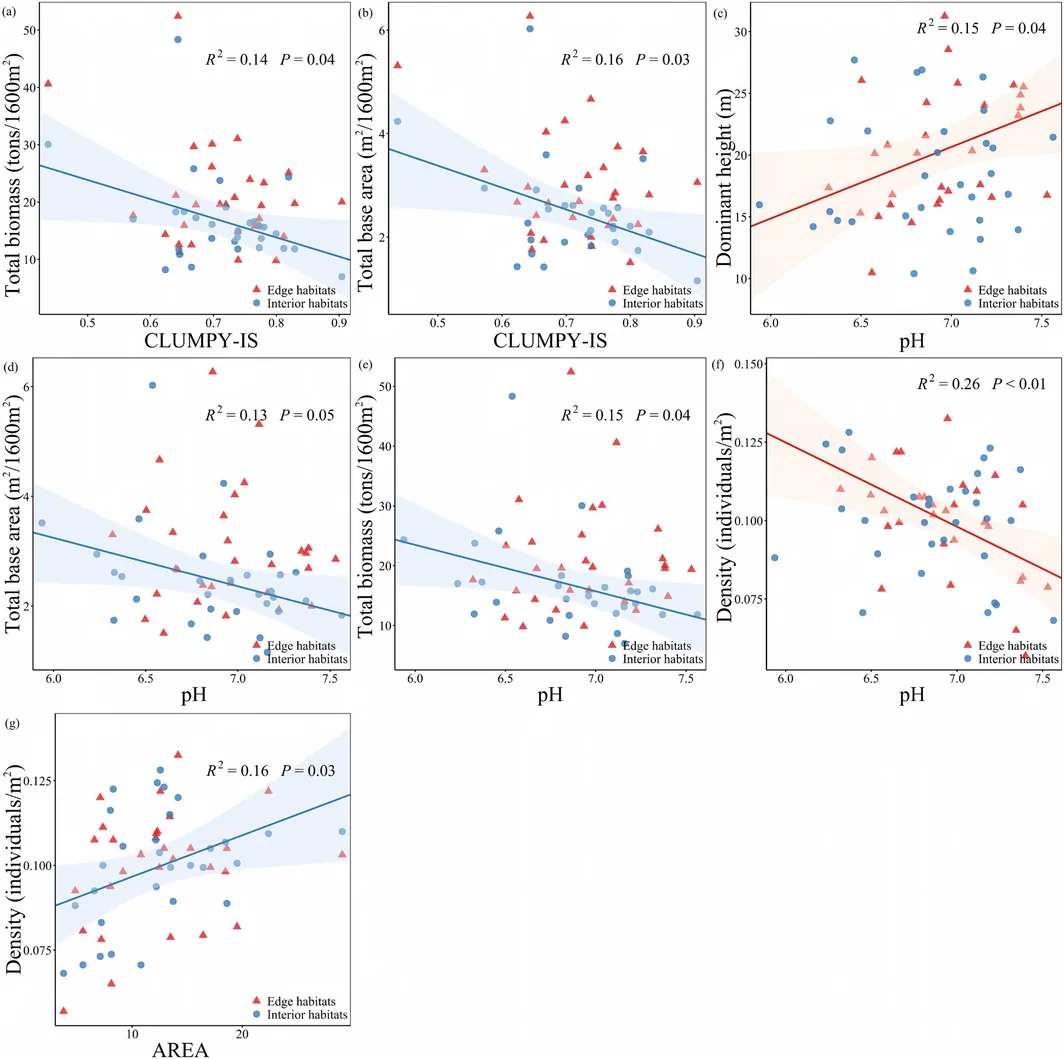
Significant regression relationships between forest structure indices and fragmentation indices or soil properties (*p* < 0.05).

The analysis of influencing factors revealed distinct associations between forest structure and fragmentation indices as well as soil properties. Regarding fragmentation metrics, both TB and TBA in the interior habitat showed significant negative correlations with the CLUMPY‐IS (Figure 3a,b). In addition, tree density in the interior was positively correlated with patch area (Figure 3g). In terms of soil properties, soil pH was positively correlated with dominant height but negatively correlated with tree density in the edge habitats (Figure 3c,f). Both TB and TBA in the interior habitats were negatively correlated with soil pH (Figure 3d,e).

### 3.3 Relationship Between Plant Diversity and Forest Structure

**FIGURE 4:**
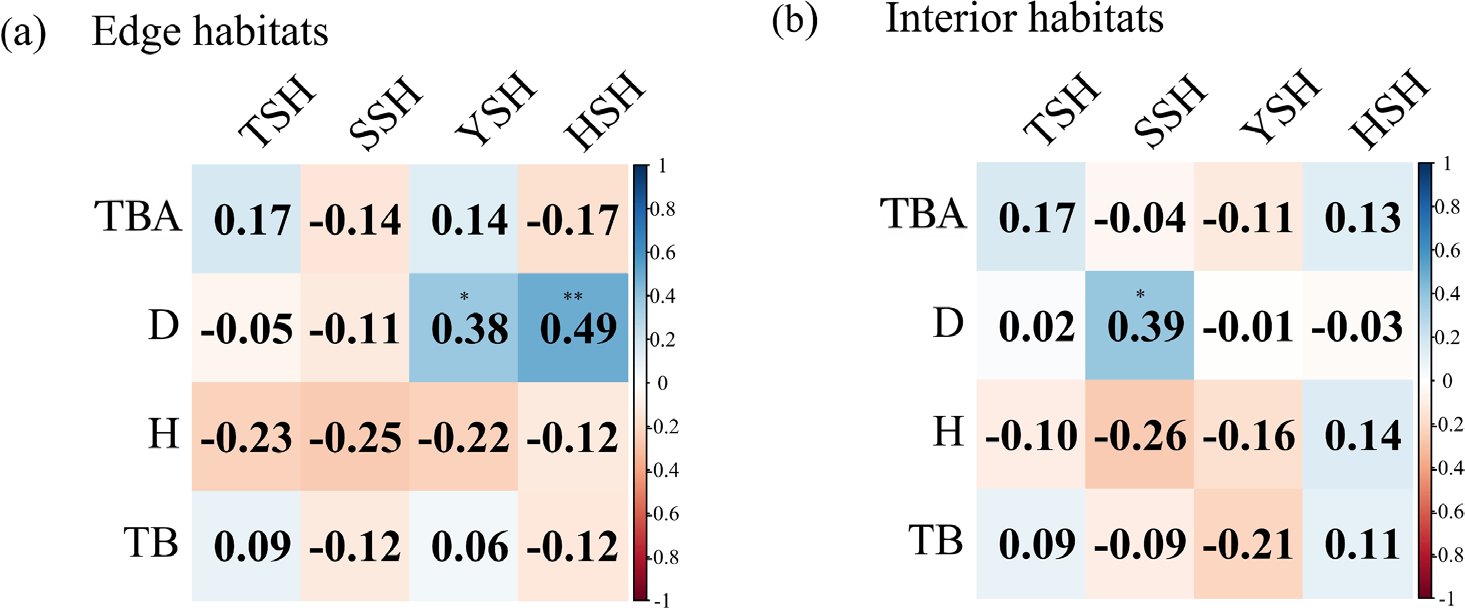
Associations between forest structure and plant diversity based on Spearman's rank correlation. *p < 0.05, **p < 0.01.

Correlation analysis revealed significant positive relationships between tree density and the diversity of different vegetation types, with distinct patterns observed between habitats. In the edge habitat, tree density was positively correlated with the Shannon diversity of saplings/seedlings and herbs (Figure 4a). Within the interior habitat, tree density showed a significant positive correlation with shrub Shannon diversity (Figure 4b).

To avoid multicollinearity, three predictors—TB, IER, and PIS—with variance inflation factors (VIF) > 4 were excluded prior to the RDA. The full VIF table can be found in Table S3. Results from both the RDA (Figure S2) and variance partitioning (Table 1) indicated that the composition of adult trees was significantly influenced by fragmentation indices and soil properties (*p* < 0.01). Shrub composition was substantially affected by fragmentation, soil properties, and forest structure (*p* < 0.01). In contrast, herb composition was significantly associated with forest structure (*p* < 0.01) and soil properties (*p* < 0.05), while the composition of saplings/seedlings was primarily shaped by fragmentation indices (*p* < 0.01) and forest structure (*p* < 0.05).

**TABLE 1:** Results of variance partitioning analysis for the effects of predictors on the composition of different vegetation types.

Vegetation type	Fragmentation	Soil property	Forest structure
Adult trees			
Adjusted *R* ^2^	0.132	0.070	0.026
*F*	2.276	2.105	1.525
*p*	0.001***	0.001***	0.056
Saplings/seedings			
Adjusted *R* ^2^	0.090	0.026	0.034
*F*	1.829	1.398	1.699
*p*	0.001***	0.061	0.022*
Shrubs			
Adjusted R^2^	0.103	0.070	0.064
*F*	1.973	2.104	2.336
*p*	0.002**	0.001***	0.001***
Herbs			
Adjusted *R* ^2^	0.033	0.042	0.107
*F*	1.288	1.648	3.358
*p*	0.076	0.026*	0.001***

* *p* ≤ 0.05.

** *p* ≤ 0.01.

*** *p* ≤ 0.001.

## 4 Discussion

Our findings demonstrate that the effects of forest fragmentation on plant diversity are fundamentally governed by the strength of edge effects and the spatial configuration of the surrounding landscape (Hending et al. 2023; Magrach et al. 2012). Specifically, high edge contrast negatively impacted diversity in the interior habitat, where strong external disturbances altered microclimatic conditions and reduced diversity, especially among disturbance‐sensitive species (Blanchard et al. 2023). Conversely, in the edge habitat, herb diversity declined with increasing aggregation of adjacent impervious surfaces. These edge zones are directly exposed to external stressors, and concentrated imperviousness imposes regional‐scale pressures that directly filter out non‐tolerant herbaceous plants (Hu et al. 2021). Additionally, greater patch shape complexity enhanced seedling and sapling diversity at the edges. Complex shapes create longer boundaries, which expand the extent of edge habitats and increase microenvironmental heterogeneity, as a result facilitating the establishment of a wider array of saplings/seedlings (Zambrano et al. 2020).

The TBA, dominant tree height, and TB were significantly greater in forest edges than in the interior (Cázares et al. 2020; Meeussen et al. 2021). This pattern can be attributed to the ample light availability in edge habitats, which stimulates the rapid growth of light‐demanding and fast‐growing pioneer species (De Pauw et al. 2024). As a result, these areas exhibit enhanced structural development and biomass accumulation over the short term (Bruna and de Andrade 2011). However, such increases often come at the cost of simplified species composition and potential long‐term declines in ecosystem stability (Wang and Yang 2022). This trade‐off highlights the dual role of edge effects in simultaneously promoting tree growth and undermining community diversity (Li, Zhao, et al. 2018). Besides, selective retention of large trees at margins, differential logging history, or survivor bias could also contribute to this structural advantage at edges.

The forest structure in the interior habitat is particularly sensitive to fragmentation indicators. Both TB and basal area in the interior showed a significant negative correlation with the clumpiness of impervious surfaces. This relationship can be explained by the extensive contiguous urban areas associated with high imperviousness, which generate regional stressors such as heat islands and pollution (Meineke et al. 2016; Zhao et al. 2025). These pressures persistently infiltrate the forest core, suppressing tree growth. Meanwhile, tree density in the interior was positively correlated with patch area (Santana et al. 2021). Larger patches provide more extensive core habitat, which better buffers against edge effects and creates safer conditions for tree regeneration and survival, and so supports higher population densities (Pincheira‐Ulbrich et al. 2009; Yamaura et al. 2008).

The relationship between tree density and plant diversity exhibited distinct patterns in remnant forest edge versus interior habitats. In the edge habitat adjacent to the urban matrix, higher tree density significantly promoted the diversity of saplings/seedlings and herbaceous plants by forming a physical buffer, improving microclimatic conditions, and creating heterogeneous microhabitats (Hofmeister et al. 2019). In contrast, within the stable interior habitat, the shaded environment resulting from high tree density favored shrub diversity (Balandier et al. 2022), as shrubs—being more shade‐tolerant and equipped with woody stems and deeper root systems—are better adapted to survive and compete under lowlight conditions (Valladares and Niinemets 2008).

The influence of soil properties on forest plant diversity and structure exhibits clear habitat specificity. In terms of diversity, within the interior habitat, SOC promoted the diversity of herbaceous plants and juveniles by improving soil structure and fertility (Spohn et al. 2023). However, elevated nitrogen levels appeared to intensify competitive exclusion among herbaceous species, by doing so reducing diversity (He et al. 2021; Martini et al. 2020). In the edge habitat, abundant soil resources intensified understory competition, which suppressed saplings/seedlings regeneration (Gruber et al. 2018), whereas increased phosphorus directly enhanced herb diversity (Li et al. 2013). Regarding forest structure, in edge habitats, soil pH facilitated vertical tree growth by optimizing nutrient availability (Yasin et al. 2014), but also led to reduced tree density due to intensified competition among individuals (Medina‐Vega et al. 2022). In contrast, within the interior habitat, higher soil pH was negatively correlated with biomass and basal area, potentially reflecting its association with certain conditions unfavorable for tree growth (Fang et al. 2024).

The composition of different vegetation strata in remnant forests is shaped by distinct environmental drivers. The current fragmentation pattern is a direct legacy of historical land use. Adult trees, representing establishment and survival over past decades, are thus influenced both by fragmentation metrics reflecting historical patterns and by soil properties that define foundational habitat conditions (Laurance et al. 2018). Shrub communities occupy a transitional position, making them sensitive to influences from the overlying forest structure, the underlying soil environment, and the surrounding fragmentation context (Landuyt et al. 2019). The herb layer, being the ground level stratum, is highly responsive to changes in the forest micro‐environment, which is primarily determined by forest structure. Moreover, with their shallow root systems, herbaceous plants react rapidly to moisture and nutrient conditions in the topsoil. The composition of saplings/seedlings is mainly controlled by fragmentation indices, which affect seed dispersal, and by forest structure, which critically determines the success of seedling establishment (Borah and Beckman 2024).

Based on the findings of this study, the following management recommendations are proposed for urban remnant forests. First, further reduction in remnant patch area should be prevented, as larger patches help enhance tree density and promote understory plant diversity. Second, multilayered buffer zones should be established to soften the stark edges of forest patches, since high edge contrast reduces herb and shrub diversity. Third, the encirclement of forests by contiguous urban expansion should be restricted, as increased clumpiness of impervious surfaces around patches impairs forest structure and plant diversity.

While this study systematically reveals important relationships between landscape patterns and both plant diversity and forest structure in urban remnant forests, its conclusions should be interpreted within the context of the following limitations. First, the use of a single 1‐km landscape scale may not capture the heterogeneity of ecological responses across different spatial scales. Second, the reliance on cross‐sectional data limits the ability to analyze dynamic ecological processes and time‐lag effects. Although soil factors were considered, the study did not include all potential covariates, such as human activity intensity and pest or disease pressure. Despite these limitations, this research provides a valuable empirical foundation for understanding fragmentation effects in urban remnant forests and highlights the need for future multi‐scale, long‐term, and multifactorial studies.

## 5 Conclusion

This study elucidates the mechanisms through which urbanization‐driven fragmentation influences plant diversity and forest structure in remnant forest patches. It demonstrates that reduced patch area, high edge contrast, and high clumpiness of surrounding impervious surfaces negatively affect the maintenance of plant diversity and forest structural complexity. Furthermore, significant positive relationships were detected between tree density and plant diversity, and the composition of understory vegetation, including saplings/seedlings, shrubs, and herbs, was significantly influenced by forest structural attributes. These findings highlight the tight coupling between forest structure and understory communities in fragmented urban landscapes, and offer new targets for the management of urban remnant forests. Future research should refine this ecological model through multi‐scale dynamic monitoring, advancing urban ecological conservation from pattern‐based planning to process‐oriented management.

## Author Contributions

**Linfeng Zhou:** data curation (lead), investigation (lead), methodology (lead), software (lead), validation (lead), visualization (lead), writing – original draft (lead), writing – review and editing (lead). **Bingxia Zhang:** investigation (equal), methodology (equal), writing – review and editing (equal). **Mengli He:** investigation (equal), methodology (equal), writing – review and editing (equal). **Minglu Guo:** investigation (equal), methodology (equal), writing – review and editing (equal). **Jingyi Yang:** conceptualization (lead), data curation (supporting), funding acquisition (lead), methodology (equal), project administration (lead), resources (lead), supervision (lead), validation (supporting), visualization (supporting), writing – review and editing (equal).

## Ethics Statement

The authors have nothing to report.

## Conflicts of Interest

The authors declare no conflicts of interest.

## Supporting information


**Table S1:** Parameters of the biomass equation.
**Table S2:** Global Moran's *I* test results for spatial autocorrelation in the response variables.
**Table S3:** Stepwise variance inflation factors (VIF) for predictor variables during variable selection for redundancy analysis (RDA).
**Figure S1:** Nonmetric multidimensional scaling (NMDS) ordination showing the grouping of different plant types.
**Figure S2:** RDA triplot illustrating the associations between predictor variables and plant compositional dissimilarity.

## Data Availability

The data for this study are available via the Mendeley Data Repository https://doi.org/10.17632/38b2mdbk6w.2.
