# Supplementary material for: Multilevel Impacts of Habitat Fragmentation on Plant Diversity and Structure in Urban Remnant Forests
Source: Ecol Evol. 2026 Aug 28;16(9):e74246. doi: 10.1002/ece3.74246 (PMC13522934; doi:10.1002/ece3.74246)
Supplement: Supplementary file 1 [file ECE3-16-e74246-s001.docx]

**Supplementary Materials**

Table S1 Parameters of the biomass equation

| Latin Name | A0 | A1 | A2 | B0 | B1 | B2 |
| --- | --- | --- | --- | --- | --- | --- |
| Quercus fabri | 0.13 | 1.83 | 0.71 | 0.11 | 2.08 | 0.28 |
| Meliosma rigida | 0.19 | 2.33 |  | 0.06 | 2.16 |  |
| Platycladus orientalis | 0.10 | 1.91 | 0.62 | 0.04 | 1.77 | 0.64 |
| Sassafras tzumu | 0.14 | 2.33 |  | 0.06 | 2.16 |  |
| Platyosprion platycarpum | 0.20 | 2.33 |  | 0.06 | 2.16 |  |
| Robinia pseudoacacia | 0.20 | 2.33 |  | 0.06 | 2.16 |  |
| Kalopanax septemlobus | 0.19 | 2.33 |  | 0.06 | 2.16 |  |
| Ilex macrocarpa | 0.19 | 2.33 |  | 0.06 | 2.16 |  |
| Quercus macrocarpa | 0.13 | 1.83 | 0.71 | 0.11 | 2.08 | 0.28 |
| Itea yunnanensis | 0.19 | 2.33 |  | 0.06 | 2.16 |  |
| Ilex chinensis | 0.19 | 2.33 |  | 0.06 | 2.16 |  |
| Eucommia ulmoides | 0.19 | 2.33 |  | 0.06 | 2.16 |  |
| Rhamnella martini | 0.14 | 2.33 |  | 0.06 | 2.16 |  |
| Carpinus turczaninovii | 0.06 | 2.11 | 0.52 | 0.03 | 2.08 | 0.28 |
| Liquidambar formosana | 0.09 | 2.26 | 0.30 | 0.12 | 2.42 | -0.40 |
| Broussonetia papyrifera | 0.13 | 2.33 |  | 0.06 | 2.16 |  |
| Cornus wilsoniana | 0.19 | 2.33 |  | 0.06 | 2.16 |  |
| Photinia bodinieri | 0.19 | 2.33 |  | 0.06 | 2.16 |  |
| Prunus laurocerasus | 0.19 | 2.33 |  | 0.06 | 2.16 |  |
| Betula davurica | 0.06 | 2.11 | 0.52 | 0.03 | 2.08 | 0.28 |
| Meliosma oldhamii | 0.19 | 2.33 |  | 0.06 | 2.16 |  |
| Cleyera japonica | 0.12 | 2.06 | 0.38 | 0.08 | 2.32 | -0.24 |
| Camphora bodinieri | 0.14 | 2.33 |  | 0.06 | 2.16 |  |
| Platycarya strobilacea | 0.19 | 2.33 |  | 0.06 | 2.16 |  |
| Styphnolobium japonicum | 0.20 | 2.33 |  | 0.06 | 2.16 |  |
| Dalbergia hupeana | 0.20 | 2.33 |  | 0.06 | 2.16 |  |
| Populus × canadensis | 0.06 | 2.05 | 0.59 | 0.02 | 2.24 | 0.09 |
| Cornus macrophylla | 0.19 | 2.33 |  | 0.06 | 2.16 |  |
| Pyrus | 0.19 | 2.33 |  | 0.06 | 2.16 |  |
| Castanea mollissima | 0.13 | 1.18 | 0.71 | 0.11 | 2.08 | 0.28 |
| Melia azedarach | 0.19 | 2.33 |  | 0.06 | 2.16 |  |
| Betula luminifera | 0.06 | 2.11 | 0.52 | 0.03 | 2.08 | 0.28 |
| Koelreuteria paniculata | 0.13 | 2.33 |  | 0.06 | 2.16 |  |
| Quercus acutissima | 0.13 | 1.81 | 0.71 | 0.11 | 2.08 | 0.28 |
| Pinus massoniana | 0.07 | 2.09 | 0.50 | 0.01 | 2.74 | -0.08 |
| Castanea seguinii | 0.13 | 1.18 | 0.71 | 0.11 | 2.08 | 0.28 |
| Ligustrum lucidum | 0.19 | 2.33 |  | 0.06 | 2.16 |  |
| Eriobotrya japonica | 0.19 | 2.33 |  | 0.06 | 2.16 |  |
| Celtis sinensis | 0.10 | 1.99 | 0.49 | 0.04 | 2.06 | 0.34 |
| Toxicodendron vernicifluum | 0.19 | 2.33 |  | 0.06 | 2.16 |  |
| Quercus glauca | 0.13 | 1.81 | 0.71 | 0.11 | 2.08 | 0.28 |
| Albizia kalkora | 0.20 | 2.33 |  | 0.06 | 2.16 |  |
| Cunninghamia lanceolata | 0.07 | 2.02 | 0.48 | 0.02 | 2.53 | -0.01 |
| Celtis julianae | 0.10 | 1.99 | 0.49 | 0.04 | 2.06 | 0.34 |
| Tetradium ruticarpum | 0.19 | 2.33 |  | 0.06 | 2.16 |  |
| Firmiana simplex | 0.13 | 2.33 |  | 0.06 | 2.16 |  |
| Camptotheca acuminata | 0.13 | 2.33 |  | 0.06 | 2.16 |  |
| Eurya nitida | 0.13 | 2.33 |  | 0.06 | 2.16 |  |
| Toona sinensis | 0.19 | 2.33 |  | 0.06 | 2.16 |  |
| Lindera communis | 0.14 | 2.33 |  | 0.06 | 2.16 |  |
| Populus adenopoda | 0.06 | 2.05 | 0.59 | 0.02 | 2.24 | 0.09 |
| Rhus chinensis | 0.19 | 2.33 |  | 0.06 | 2.16 |  |
| Morella rubra | 0.19 | 2.33 |  | 0.06 | 2.16 |  |
| Diospyros kaki var. silvestris | 0.19 | 2.33 |  | 0.06 | 2.16 |  |
| Euscaphis japonica | 0.19 | 2.33 |  | 0.06 | 2.16 |  |
| Ginkgo biloba | 0.19 | 2.33 |  | 0.06 | 2.16 |  |
| Prunus pseudocerasus | 0.19 | 2.33 |  | 0.06 | 2.16 |  |
| Camellia oleifera | 0.12 | 2.06 | 0.38 | 0.08 | 2.32 | -0.24 |
| Torricellia angulata var. intermedia | 0.13 | 2.33 |  | 0.06 | 2.16 |  |
| Ulmus pumila | 0.10 | 1.99 | 0.49 | 0.04 | 2.06 | 0.33 |
| Carpinus pubescens | 0.06 | 2.11 | 0.52 | 0.03 | 2.08 | 0.28 |
| Camphora glandulifera | 0.14 | 2.33 |  | 0.06 | 2.16 |  |
| Gleditsia sinensis | 0.20 | 2.33 |  | 0.06 | 2.16 |  |
| Quercus augustinii | 0.13 | 1.81 | 0.71 | 0.11 | 2.08 | 0.28 |
| Camphora officinarum | 0.14 | 2.33 |  | 0.06 | 2.16 |  |
| Catalpa ovata | 0.19 | 2.33 |  | 0.06 | 2.16 |  |
| Cercis chinensis | 0.19 | 2.33 |  | 0.06 | 2.16 |  |
| Prunus cerasifera cv. Atropurpurea | 0.19 | 2.33 |  | 0.06 | 2.16 |  |

Table S2 Global Moran’s I test results for spatial autocorrelation in the response variables.

| Habitat type | Variable | Moran’s I | Z | p |
| --- | --- | --- | --- | --- |
| Interior habitat | TBA | -0.161 | -1.602 | 0.109 |
|  | D | 0.018 | 0.565 | 0.572 |
|  | H | 0.061 | 1.017 | 0.309 |
|  | TB | -0.193 | -1.906 | 0.057 |
|  | TSH | 0.063 | 1.053 | 0.292 |
|  | SSH | -0.104 | -0.737 | 0.461 |
|  | YSH | -0.128 | -1.017 | 0.309 |
|  | HSH | -0.053 | -0.201 | 0.841 |
| Edge habitat | TBA | -0.172 | -1.531 | 0.126 |
|  | D | 0.171 | 2.230 | 0.026 |
|  | H | 0.011 | 0.487 | 0.626 |
|  | TB | -0.155 | -1.396 | 0.163 |
|  | TSH | 0.014 | 0.511 | 0.609 |
|  | SSH | -0.095 | -0.646 | 0.518 |
|  | YSH | 0.134 | 1.831 | 0.067 |
|  | HSH | 0.050 | 0.904 | 0.366 |

Table S3 Stepwise variance inflation factors (VIF) for predictor variables during variable selection for redundancy analysis (RDA).

| Variable | Step 1 (All variables) | Step 2 (TB removed) | Step 3 (IER removed) | Final (PIS removed) |
| --- | --- | --- | --- | --- |
| AREA | 5.12 | 5.12 | 1.60 | 1.50 |
| TIME | 3.12 | 2.84 | 2.77 | 1.60 |
| FRAC | 6.77 | 6.64 | 1.68 | 1.68 |
| ECON | 2.83 | 2.80 | 2.70 | 2.70 |
| CLUMPY-IS | 4.56 | 4.48 | 4.13 | 3.44 |
| IER | 9.42 | 9.31 | – | – |
| PFC | 1.90 | 1.86 | 1.85 | 1.46 |
| PIS | 4.28 | 3.93 | 3.86 | – |
| CLUMPY-FC | 2.44 | 2.30 | 2.25 | 2.22 |
| TOC | 1.54 | 1.54 | 1.52 | 1.46 |
| TN | 2.06 | 2.06 | 2.06 | 2.03 |
| TP | 2.45 | 2.36 | 2.35 | 2.33 |
| PH | 1.88 | 1.87 | 1.85 | 1.84 |
| TBA | 22.66 | 1.89 | 1.70 | 1.70 |
| D | 1.75 | 1.74 | 1.73 | 1.73 |
| H | 1.58 | 1.56 | 1.47 | 1.47 |
| TB | 22.72 | – | – | – |

Note: Variables with VIF > 4 were sequentially removed. “–” indicates that the variable had already been excluded at that step. The final set of predictors (VIF < 4) was used in RDA.

Fig. S1. Non-metric multidimensional scaling (NMDS) ordination showing the grouping of different plant types

Fig. S2. RDA triplot illustrating the associations between predictor variables and plant compositional dissimilarity.
